# Investigation of Public Acceptance of Misinformation Correction in Social Media Based on Sentiment Attributions: Infodemiology Study Using Aspect-Based Sentiment Analysis

**DOI:** 10.2196/50353

**Published:** 2024-08-16

**Authors:** Ning Ma, Guang Yu, Xin Jin

**Affiliations:** 1 School of Management Harbin Institute of Technology Harbin China; 2 School of Social Sciences Harbin Institute of Technology Harbin China

**Keywords:** misinformation correction, sentiment attribution, public acceptance, public sentiments, aspect-based sentiment analysis, pretraining model

## Abstract

**Background:**

The proliferation of misinformation on social media is a significant concern due to its frequent occurrence and subsequent adverse social consequences. Effective interventions for and corrections of misinformation have become a focal point of scholarly inquiry. However, exploration of the underlying causes that affect the public acceptance of misinformation correction is still important and not yet sufficient.

**Objective:**

This study aims to identify the critical attributions that influence public acceptance of misinformation correction by using attribution analysis of aspects of public sentiment, as well as investigate the differences and similarities in public sentiment attributions in different types of misinformation correction.

**Methods:**

A theoretical framework was developed for analysis based on attribution theory, and public sentiment attributions were divided into 6 aspects and 11 dimensions. The correction posts for the 31 screened misinformation events comprised 33,422 Weibo posts, and the corresponding Weibo comments amounted to 370,218. A pretraining model was used to assess public acceptance of misinformation correction from these comments, and the aspect-based sentiment analysis method was used to identify the attributions of public sentiment response. Ultimately, this study revealed the causality between public sentiment attributions and public acceptance of misinformation correction through logistic regression analysis.

**Results:**

The findings were as follows: First, public sentiments attributed to external attribution had a greater impact on public acceptance than those attributed to internal attribution. The public associated different aspects with correction depending on the type of misinformation. The accuracy of the correction and the entity responsible for carrying it out had a significant impact on public acceptance of misinformation correction. Second, negative sentiments toward the media significantly increased, and public trust in the media significantly decreased. The collapse of media credibility had a detrimental effect on the actual effectiveness of misinformation correction. Third, there was a significant difference in public attitudes toward the official government and local governments. Public negative sentiments toward local governments were more pronounced.

**Conclusions:**

Our findings imply that public acceptance of misinformation correction requires flexible communication tailored to public sentiment attribution. The media need to rebuild their image and regain public trust. Moreover, the government plays a central role in public acceptance of misinformation correction. Some local governments need to repair trust with the public. Overall, this study offered insights into practical experience and a theoretical foundation for controlling various types of misinformation based on attribution analysis of public sentiment.

## Introduction

### Background

Scholarly interest in researching misinformation dissemination has been extensive [1,2], particularly given the exacerbation of this issue since the COVID-19 pandemic. Such misinformation encompasses a range of forms, including the spread of health misinformation [3], fake news [4], conspiracy theories [5], and other types of COVID-19 misinformation [6]. The spread of misinformation can significantly hinder individuals’ access to accurate information and may exacerbate psychological distress [7]. In addition, the public may lose faith in the government, leading to increased fear and anxiety [8]. The resulting infodemic has heightened concerns about the adverse effects of misinformation [9,10] and prompted greater research attention toward misinformation and infodemic governance [11].

Research on how to mitigate the spread of misinformation has resulted in several recommended interventions [12]. The intervention and prevention strategies for misinformation are also key research points [13]. Fact checking was believed to be an effective approach in reducing the spread of misinformation, but it often requires extensive coordination to improve public resistance [14]. Applying warning tags to social media posts could also potentially reduce sharing behaviors [15], but the credibility of untagged posts could be potentially enhanced [16]. Inaccurate tagging can also aggravate the spread of misinformation, making warning tags ineffective [17]. Another effective approach is to post correction information that directly addresses false facts or claims [18], with authoritative experts playing a prominent role in correction communication [9,19,20]. Using social media platforms and the structure of social networks to block misinformation is also an effective approach [21], and correcting information in real time through social media platforms is another strategy [22].

Preventive measures are also necessary to combat misinformation among the public [12]. The most common framework for implementing preventive interventions is the psychological inoculation theory [23]; it had lasting effects in responding to misinformation about COVID-19 [24]. Furthermore, educational preventive measures are thought to be the most effective [25,26], and personal awareness of misinformation is equally significant [27]. To implement reliable preventive measures on a large scale, we need more professionals with advanced knowledge and convergence of many efforts to yield reliable results [12]. Therefore, government policy support is essential [28], and a clear legal framework on misinformation needs to be established promptly [29]. However, both debunking [30] and prebunking, which refers to prophylactic interventions [23], have limitations and may not be as effective as anticipated [12]. While scholars have proposed suggestions to improve the correction effect [31], sometimes misinformation correction can even backfire [32].

Although researchers have made concerted efforts to correct misinformation on social networks, the results have been suboptimal in many cases [33]. This not only perpetuates misinformation but also risks public resentment [34], undermining the persuasive power of subsequent corrections. Current research on evaluating the effectiveness of misinformation correction through social media has analyzed the impact of various post characteristics on correction outcomes [35]. As emotional information can provide valuable insights into understanding the public perception of misinformation [36-39], more research is needed on how public sentiment can be leveraged to analyze the reasons for poor correction effectiveness. Analyzing public sentiment can help identify public attitudes toward misinformation [34,40], including the effectiveness of correction. Considering the limitations regarding objectivity and timeliness of using questionnaires to assess public attitudes toward misinformation and correction [41], social media data can be used more objectively to analyze public acceptance of misinformation correction [42]. Governments can also use public sentiment analysis to combat misinformation and inappropriate sharing behaviors [43].

### Objectives

It is vital to understand how to evaluate public acceptance of misinformation correction using public sentiment analysis on social media. To address this research goal, this study focused on misinformation correction during the COVID-19 pandemic and mining the key factors influencing public acceptance of correction. Specifically, the following three research questions were answered in this study:

1. How to characterize public acceptance of misinformation correction and different aspects of public sentiment? (Research question 1)
2. What are the differences in the impact of various public sentiment attributions on the correction of different types of misinformation? (Research question 2)
3. Which attribution of public sentiment is more critical to shaping public acceptance of correction of different types of misinformation? (Research question 3)

To comprehensively explore the aforementioned questions, this study collected public interaction data on the Weibo platform pertaining to important misinformation dissemination events related to COVID-19 since the outbreak. First, aspect-based sentiment analysis (ABSA) was performed to attribute public sentiments to multiple dimensions drawing on attribution theory. Second, the study explored how public acceptance of misinformation correction related to public sentiment attributions in different events from the public perspective. Finally, the significant degree of attribution results obtained through public sentiment response revealed the specific reasons for public attitudes. Consequently, this study provides theoretical support and practical evidence for developing correction strategies for misinformation, which could positively impact the formulation of correction communication measures.

## Methods

### Research Design and Framework

**Figure 1:**
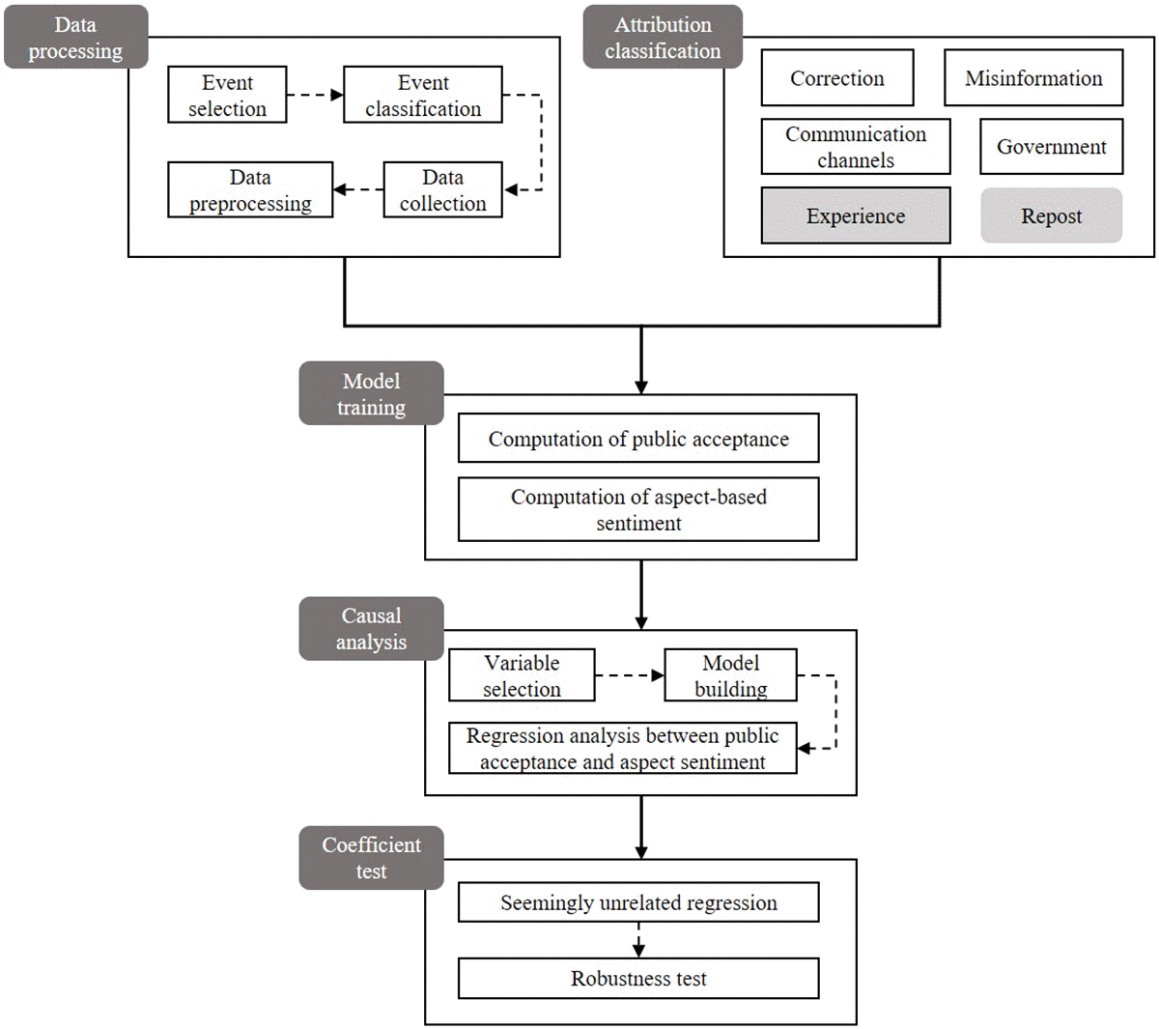
Research and analysis framework of public acceptance of misinformation correction based on attribution theory. The framework includes the specific steps of data collection, model training, causality analysis, and other processes.

This study proposed a research framework, as depicted in Figure 1, based on attribution theory to evaluate public acceptance of misinformation correction by analyzing the attribution results of public sentiment response to correction.

The underlying reasons for the ineffectiveness of misinformation correction as perceived by the public were elucidated by this framework. By examining various types of misinformation dissemination events, we categorized and analyzed key public sentiment attributions that contribute to public acceptance of misinformation correction in different situations. Furthermore, we considered public views that are not directly related to correction and whose role in analyzing the efficacy of correction cannot be ignored.

### Event Selection and Data Collection

Weibo is a prominent social media platform in China that has been extensively studied in relation to public opinion [42]. One noteworthy feature of Weibo is the Weibo Refuting Rumor account, which serves to clarify and correct rumors that circulate on the web. This study used the posts from the Weibo Refuting Rumors account to screen 31 misinformation events related to COVID-19 that occurred between January 26, 2020, and April 14, 2022. The corresponding correction posts and their comments were obtained through data support from Zhiwei Data Sharing Platform. In total, the correction posts for the 31 events comprised 33,422 Weibo posts, and the corresponding Weibo comments amounted to 370,218. This study analyzed these comments to investigate the attributions that influenced public attitudes regarding misinformation correction.

Current research on misinformation, particularly related to COVID-19, focuses on categorizing it based on content. One such classification method through fact checking divided misinformation into 6 categories, which included treatment, conspiracy, government measures, vaccines, number of cases, and others [38]. Drawing from this categorization, this study categorized 31 misinformation events related to COVID-19 into 4 main categories through fact checking and examining the public opinion on social networks in China: prevention and treatment, conspiracy, government measures, and COVID-19 development. (Specific event information and classification can be found in Multimedia Appendix 1.)

### Main Components of Sentiment Attributions in Misinformation Correction

Public sentiment responses to misinformation correction can originate from various sources [44]. According to attribution theory, public perception of misinformation correction is influenced by how they attribute the causes of their sentiment response. This attribution process involves 3 dimensions: locus, stability, and controllability [45]. When the public encounters misinformation correction, the attribution of public sentiment response ultimately falls on the issue of locus of causality. At this point, whether the public sentiment responses are attributed to internal or external attributions becomes the key of the research [46,47].

This study categorized the sources of public sentiment based on the key elements of misinformation into 4 categories: the background of the event, the channel of information, the information itself, and the recipient [34]. Regarding actual misinformation correction, the background of the event corresponds to the aspect of misinformation, the channel of information refers to the communication channel aspect, the information itself pertains to the correction aspect, and the recipient is the public. The internal attribution of public sentiment pertains to the public’s own experience as the recipient, whereas other aspects of public sentiment fall under external attribution. Public sentiment responses attributed to these different aspects are associated with public acceptance of misinformation correction. Furthermore, the influence of the government on public sentiment is a crucial factor, and its response to misinformation also affects the public sentiment response [28]. According to the aforementioned theoretical analysis, public sentiments were divided into fine-grained sentiments in 6 aspects and 11 dimensions, which are detailed in Table 1 and reflect the attributions of public sentiment response.

**Table 1:** The specific description and classification of sentiment attribution into aspects.

Locus, aspect, and dimension				Specific description
**External**				
	**Correction**			
		MCB	Correction action against misinformation	
		MD	Debunker of misinformation	
	**Misinformation**			
		MMO	The main object of misinformation events	
		MSB	The spread and manufacturing of misinformation	
		DOM	Disseminators and producers of misinformation	
	**Communication channels**			
		CM	Communication media and celebrities	
	**Government**			
		GA	Official government and public authority representative	
		LG	Local government	
		TM	Treatment and response measures in misinformation events	
**Internal**				
	**Experience**			
		SP	Personal feelings and experience	
**No attribution**				
	**Repost**			
		REP	Repost	

### Ethical Considerations

The comment data used for this study are publicly accessible on the Weibo platform, and none of the data contained any details that could be used to track individual users. The authors consulted with their respective institutional review boards and were informed that no approvals were necessary.

### Computation of Public Acceptance and Public Sentiments

To evaluate public attitudes toward misinformation correction in the comments, it is crucial to extract public acceptance of the correction. As the text data used in this study were in Chinese, the Bidirectional Encoder Representations from Transformers (BERT) pretraining model based on the Chinese pretraining model of the Social Computing and Information Retrieval Research Center from the Harbin Institute of Technology was used to assess public opinions [48,49]. Specifically, comments were labeled as *tag 1* if the public recognized the correction and as *tag 0* if the public considered the correction unreliable or ineffective. In the labeling process, 2 professional researchers randomly selected 10,000 comments from all the processed Weibo comments and labeled them based on whether the public recognized the correction. The tagged text was then cross-validated by 2 professional researchers. After labeling, the data were split into validation and training sets in a 1:9 ratio, with labels of 1 representing public acceptance and 0 representing public nonacceptance. The pretraining model was then adjusted to the use environment and used for model training. The model had a good performance, and it is presented in Table 2. It was used to assess public acceptance of the correction in the remaining comments.

**Table 2:** Model performance of Bidirectional Encoder Representations from Transformers (BERT) and aspect-based sentiment analysis (ABSA) on different indicators.

Model and effectiveness indicator			Performance (%)
**BERT_Roberta**			
	Accuracy	83.21	
	Recall	81.55	
	*F*_1_-score	80.71	
**ABSA**			
	APC_accuracy^a^	87.27	
	APC_f1_score^b^	87.18	
	ATE_accuracy^c^	89.08	
	ATE_f1_score^d^	88.87	

^a^APC_accuracy: ABSA development has progressed effectively. The accuracy of aspect polarity category.

^b^APC_f1_score: The *F*_1_-score of aspect polarity category.

^c^ATE_accuracy: The accuracy of aspect term extraction.

^d^ATE_f1_score: The *F*_1_-score of aspect term extraction.

To identify the specific target objects of public sentiments in each text and analyze the attribution of public sentiment response, this study used the aspect-based sentiment computing method. ABSA is a method to analyze the sentiments of different aspects of an object in text. Unlike traditional sentiment classification, which only provides the comprehensive sentiment polarity of the text, ABSA focuses on both target extraction and target sentiment classification, making it a more fine-grained sentiment analysis method [50,51]. ABSA development has progressed effectively and has greatly improved the performance of the model, especially Chinese ABSA [52]. The most common application scenario for ABSA is in content analysis of restaurant and hotel reviews [53], where it completed the task of fine-grained sentiment analysis with 5 aspects and 18 dimensions [54]. Recently, ABSA has also been applied to extract themes and analyze relevant discourse on social media during COVID-19 [55], and other scholars have conducted aspect-based fine-grained sentiment analysis on user interaction data on social media about country image during the COVID-19 pandemic [56].

This study used ABSA involving 2 tasks: aspect term extraction and aspect polarity classification [52]. To adapt to the language environment of misinformation on Weibo, the corpus labeling rules were adjusted, and public sentiments were classified into negative and nonnegative categories. The texts were labeled according to the classification in Table 1. In total, 2 professional researchers labeled the corpus, resulting in a total of 17,629 corpora through cross-validation, which were divided into validation and training sets with a ratio of 1:9 for model training. The model performance is shown in Table 2. This model was then used to predict aspect sentiments in the remaining comments and investigate the specific attributions of public sentiments.

### Causal Analysis of Public Acceptance of Correction

This study used logistic regression analysis to investigate the particular attributions that impact public acceptance of misinformation correction. The dependent variable for this analysis was public acceptance of misinformation correction, whereas the independent variables were the number of negative and nonnegative sentiments in the text across 11 dimensions. Moreover, the study controlled for gender, number of fans, number of likes, geographical location, and user authentication identity type by incorporating them as control variables in the following logistic regression model:

Z = ln(p/[1 – p]) = b_0_ + b_1_MCB_non +b_2_MCB_neg + b_3_MCB_non + b_4_MD_neg + b_5_MMO_non + b_6_MMO_neg + b_7_MSB_non + b_8_MSB_neg + b_9_DOM_non + b_10_DOM_neg + b_11_CM_non + b_12_CM_neg + b_13_GA_non + b_14_GA_neg + b_15_LG_non + b_16_LG_neg + b_17_TM_non + b_18_TM_neg + b_19_SP_non + b_20_SP_neg + b_21_REP + b_22_GEN + b_23_FAN + b_24_LIKE + b_25_LOC + b_26_TYPE + ε_0_,

Where *p* was the probability that the public thought the correction was valid, 1 – *p* was the probability that the public thought the correction was invalid, *b_i_* was the regression coefficient of each variable, *b_0_* was the constant term, and e_0_ was the random disturbance term. The specific variable descriptions are listed in Textbox 1. As the aspect sentiment calculation for each comment was based on the number of negative or nonnegative sentiments present within that comment, each variable representing aspect sentiment included both negative and nonnegative dimensions.

**Variable names and description of logistic regression analysis.**

**Variable and description**

- MCB_non: number of nonnegative sentiments about correction action against misinformation
- MCB_neg: number of negative sentiments about correction action against misinformation
- MD_non: number of nonnegative sentiments about debunker of misinformation
- MD_neg: number of negative sentiments about debunker of misinformation
- MMO_non: number of nonnegative sentiments about the main object of misinformation events
- MMO_neg: number of negative sentiments about the main object of misinformation events
- MSB_non: number of nonnegative sentiments about the spread and manufacturing of misinformation
- MSB_neg: number of negative sentiments about the spread and manufacturing of misinformation
- DOM_non: number of nonnegative sentiments about disseminators and producers of misinformation
- DOM_neg: number of negative sentiments about disseminators and producers of misinformation
- CM_non: number of nonnegative sentiments about communication media and celebrities
- CM_neg: number of negative sentiments about communication media and celebrities
- GA_non: number of nonnegative sentiments about the official government and public authority representatives
- GA_neg: number of negative sentiments about the official government and public authority representatives
- LG_non: number of nonnegative sentiments about local government
- LG_neg: number of negative sentiments about local government
- TM_non: number of nonnegative sentiments about treatment and response measures in misinformation events
- TM_neg: number of negative sentiments about treatment and response measures in misinformation events
- SP_non: number of nonnegative sentiments about personal feelings and experience
- SP_neg: number of negative sentiments about personal feelings and experience
- REP: repost
- GEN: gender
- FAN: number of fans
- LIKE: number of likes
- LOC: the geographical location of the user’s registration
- TYPE: user authentication identity type

This study examined the primary public sentiment attributes that influenced public acceptance of misinformation correction posted on social media based on theoretical hypotheses and conceptual models. From a public sentiment perspective, this study extracted variables from various aspects while controlling for user-specific attributes such as gender, number of fans, number of likes, and geographical location. The aim was to analyze the attributions of public sentiments related to public acceptance of misinformation correction. Thus, this study sought to identify why the public perceived correction to be ineffective and provided theoretical support for relevant correction communication methods.

### Differences in Public Sentiment Attributions Among the Corrections of Different Types of Misinformation

We constructed a regression model to analyze which variables had a significant impact on public acceptance of misinformation correction. However, it remained to be tested whether the influence of the same variable on different types of misinformation could be compared through the regression coefficients. In this study, we categorized the misinformation events into 4 types: prevention and treatment (type P), conspiracy (type C), government measures (type G), and COVID-19 development (type D). The 4 types of misinformation constituted 4 subsample groups, and we tested the differences in coefficients between groups for all variables except for the control variables using coefficient tests based on the seemingly unrelated regression. Existing methods can only test the coefficient difference between 2 groups of samples at a single time, so we had to test the coefficient difference among the 4 subsample groups in pairs. This approach enabled us to assess the difference in the role of a certain aspect of public sentiment in different types of misinformation, providing strategic support for the implementation of correction communication.

## Results

### Multidimensional Public Sentiments in Misinformation Correction

We conducted statistical analysis on public acceptance of misinformation correction in all comments and public sentiment response to different aspects, including the control variables, for the entire sample data. Table 3 presents the descriptive statistics of the overall sample data. As *GEN*, *LOC*, and *TYPE* were categorical variables and *LOC* and *TYPE* had numerous categories, the specific count of each category is not listed in Table 3.

Simple repost aspect was considered as the repost behavior after recognizing the correction in this study. However, Table 3 reveals that the average level of public acceptance of misinformation correction was 0.508, indicating that slightly over half of the public perceived the correction as effective. This finding suggests that the impact of correction on the public was not sufficiently robust. In addition, the statistics of aspect sentiments demonstrated that the number of aspect sentiments expressed in each text varied. Negative sentiments toward the dimensions of government treatment and response measures could appear up to 12 times in a single comment. Some comments did not even express sentiments about a particular dimension and might have solely been reposts.

**Figure 2:**
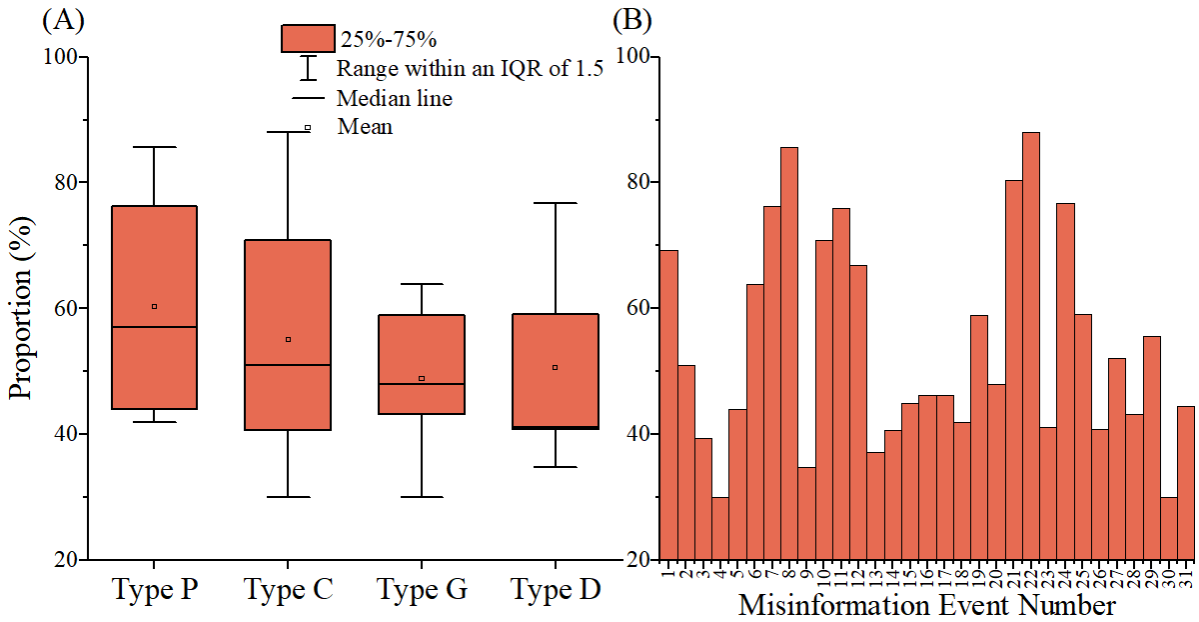
The proportion of public acceptance of correction. These 31 misinformation events related to COVID-19 occurred between January 26, 2020, and April 14, 2022. (A) Box plot of the proportion of public acceptance of correction in each type of misinformation. (B) Histogram of the proportion of public acceptance of correction in each misinformation event.

Upon examining public acceptance of misinformation correction by event type, we identified differences in the level of acceptance across different types of misinformation. As depicted in Figure 2, the correction of type P garnered the highest public acceptance, whereas the correction of types G and D had the lowest acceptance. However, public acceptance of correction of type C fluctuated greatly, suggesting that the effect of correction for type C was not consistent. Moreover, Figure 2 does not reveal any distinct pattern regarding public acceptance of correction based on the sequence of events. Therefore, overall, corrections did not yield fixed and effective outcomes.

**Figure 3:**
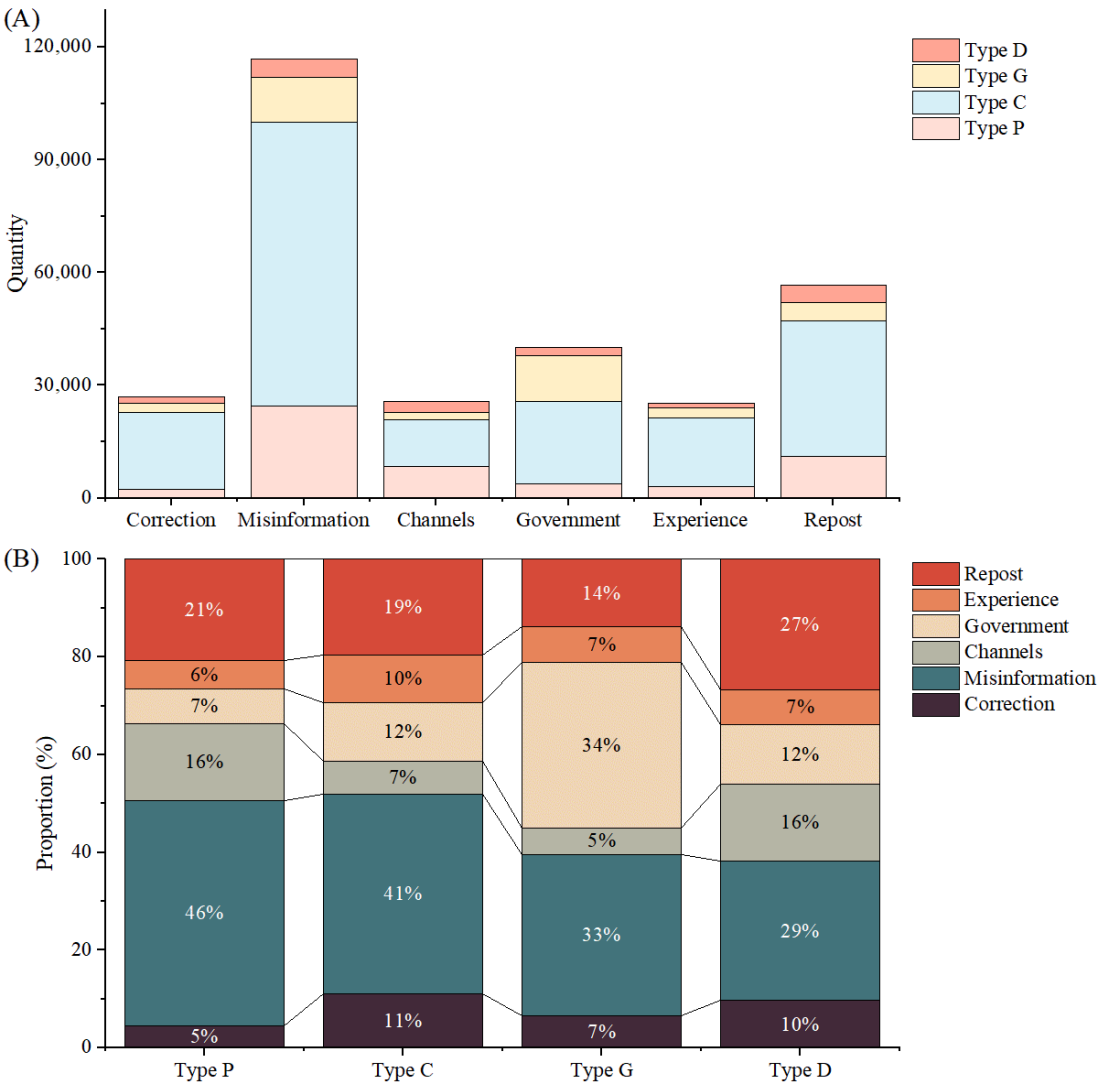
The composition of aspect sentiments in the correction of different types of misinformation. These 31 misinformation events were divided into 4 types: prevention and treatment (type P), conspiracy (type C), government measures (type G), and COVID-19 development (type D). (A) The number of public sentiments in different aspects. (B) The proportion of public sentiments in different aspects.

The findings presented in Figure 3 indicate that the comments on correction contained more sentiments regarding the aspect of misinformation, creating a noticeable gap between this and the other aspects. Aside from simple reposts, the number of sentiments in the remaining aspects was nearly equal. Furthermore, the composition of sentiments in each aspect of correction significantly varied across different types of misinformation, as evidenced by the proportion of sentiments toward correction depicted in Figure 3. For instance, comments on the correction of type G displayed significantly more sentiment toward the government compared to comments on the correction of other types. These observations indicated that there were variations in public acceptance of the correction of different types of misinformation, with marked differences in the composition of sentiments across specific aspects. Hence, conducting differentiated analyses on the correction of different types of misinformation was necessary to identify the specific reasons for poor public acceptance of correction.

**Figure 4:**
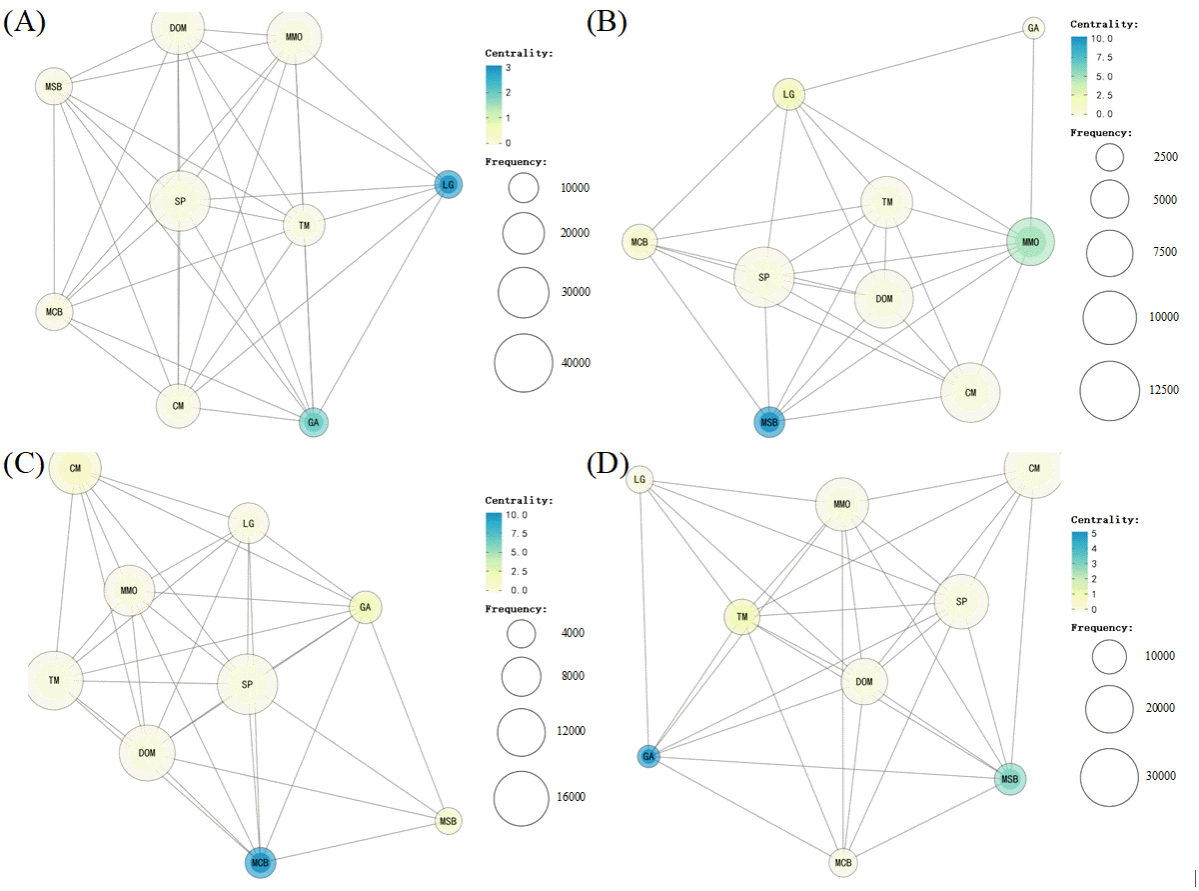
The attribution co-occurrence network of public aspect sentiment in the correction of different types of misinformation. (A) The co-occurrence network of type C. (B) The co-occurrence network of type D. (C) The co-occurrence network of type G. (D) The co-occurrence network of type P. CM: communication media and celebrities; DOM: disseminators and producers of misinformation; GA: official government and public authority representatives; LG: local government; MCB: correction action against misinformation; MMO: main object of misinformation events; MSB: spread and manufacturing of misinformation; SP: personal feelings and experience; TM: treatment and response measures in misinformation events.

Figure 4 depicts the co-occurrence network of public aspect sentiments in the correction of different types of misinformation, which highlights the disparities in public expression. The co-occurrence network illustrates the co-occurrence of different dimensions of aspect sentiments in public expression. The Jaccard coefficient served as the index for drawing the co-occurrence network, where all edges with a Jaccard coefficient of >0.1 were displayed. Our analysis revealed that the most central aspect sentiment was not necessarily the most frequent among different types of misinformation, and the central aspect sentiment in each type of misinformation network differed. For instance, in type C, the aspect sentiment of the *LG* dimension was central, whereas the aspect sentiment of *MSB* was central in type D. The aspect sentiment of *MCB* was central in type G, and the aspect sentiment of *GA* was central in type P. These differences in co-occurrence networks suggested the need for differentiated analyses of comments to uncover the reasons why the correction was not more recognized by the public.

**Table 3:** Descriptive statistics of the variables.

Variable	Observations, n	Values, mean (SD; range)
Attitudes	370,218	0.508 (0.5; 0-1)
MCB_non^a^	370,218	0.028 (0.199; 0-8)
MCB_neg^b^	370,218	0.036 (0.213; 0-8)
MD_non^c^	370,218	0.007 (0.1; 0-3)
MD_neg^d^	370,218	0.002 (0.04; 0-3)
MMO_non^e^	370,218	0.116 (0.377; 0-5)
MMO_neg^f^	370,218	0.066 (0.286; 0-7)
MSB_non^g^	370,218	0.017 (0.135; 0-5)
MSB_neg^h^	370,218	0.063 (0.275; 0-5)
DOM_non^i^	370,218	0.007 (0.088; 0-3)
DOM_neg^j^	370,218	0.047 (0.244; 0-6)
CM_non^k^	370,218	0.034 (0.2; 0-7)
CM_neg^l^	370,218	0.035 (0.22; 0-6)
GA_non^m^	370,218	0.019 (0.154; 0-6)
GA_neg^n^	370,218	0.014 (0.127; 0-5)
LG_non^o^	370,218	0.013 (0.122; 0-6)
LG_neg^p^	370,218	0.009 (0.101; 0-7)
TM_non^q^	370,218	0.024 (0.167; 0-4)
TM_neg^r^	370,218	0.029 (0.181; 0-12)
SP_non^s^	370,218	0.042 (0.209; 0-3)
SP_neg^t^	370,218	0.026 (0.164; 0-4)
REP^u^	370,218	0.153 (0.362; 0-3)
GEN^v^	370,218	1.485 (0.516; 1-3)
FAN^w^	370,218	35,210.49 (1,458,389; 0-2.31×10^8^)
LIKE^x^	370,218	7.264 (484.681; 0-173,209)
LOC^y^	370,218	20.388 (9.079; 1-36)
TYPE^z^	370,218	5.732 (4.674; 1-11)

^a^MCB_non: number of nonnegative sentiments about correction action against misinformation.

^b^MCB_neg: number of negative sentiments about correction action against misinformation.

^c^MD_non: number of nonnegative sentiments about debunker of misinformation.

^d^MD_neg: number of negative sentiments about debunker of misinformation.

^e^MMO_non: number of nonnegative sentiments about the main object of misinformation events.

^f^MMO_neg: number of negative sentiments about the main object of misinformation events.

^g^MSB_non: number of nonnegative sentiments about the spread and manufacturing of misinformation.

^h^MSB_neg: number of negative sentiments about the spread and manufacturing of misinformation.

^i^DOM_non: number of nonnegative sentiments about disseminators and producers of misinformation.

^j^DOM_neg: number of negative sentiments about disseminators and producers of misinformation.

^k^CM_non: number of nonnegative sentiments about communication media and celebrities.

^l^CM_neg: number of negative sentiments about communication media and celebrities.

^m^GA_non: number of nonnegative sentiments about the official government and public authority representatives.

^n^GA_neg: number of negative sentiments about the official government and public authority representatives.

^o^LG_non: number of nonnegative sentiments about local government.

^p^LG_neg: number of negative sentiments about local government.

^q^TM_non: number of nonnegative sentiments about treatment and response measures in misinformation events.

^r^TM_neg: number of negative sentiments about treatment and response measures in misinformation events.

^s^SP_non: number of nonnegative sentiments about personal feelings and experience.

^t^SP_neg: number of negative sentiments about personal feelings and experience.

^u^REP: repost.

^v^GEN: gender.

^q^FAN: number of fans.

^x^LIKE: number of likes.

^y^LOC: the geographical location of the user’s registration.

^z^TYPE: user authentication identity type.

### Causality Between Public Acceptance of Misinformation Correction and Attributions of Public Sentiment

**Figure 5:**
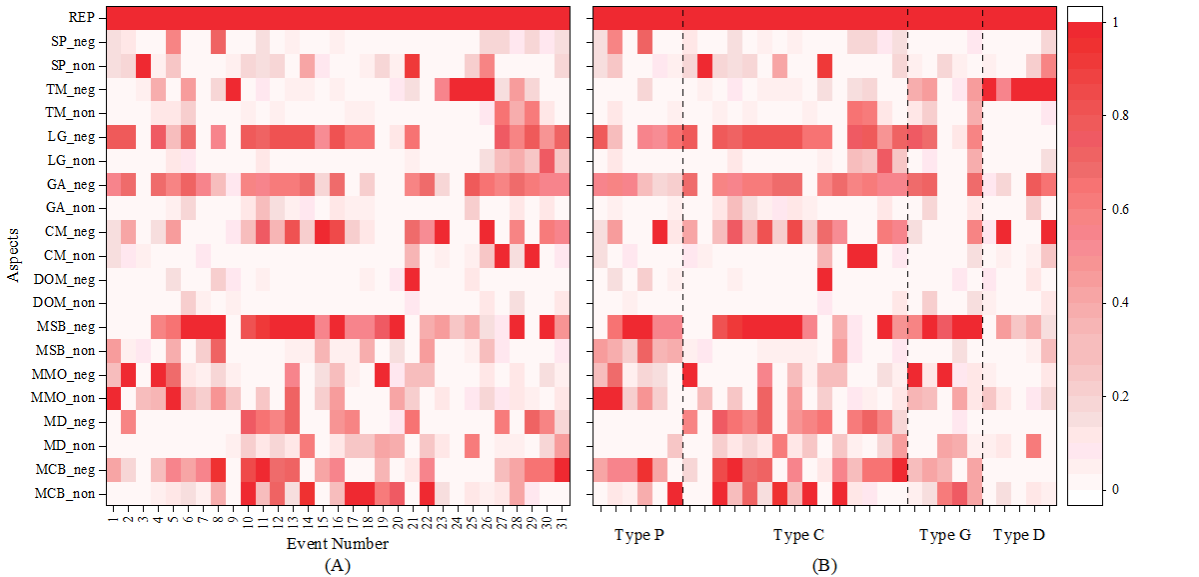
The relative significance intensity of different aspects of public sentiment. The redder the color block, the higher the relative significance intensity. (A) The relative significance intensity in each misinformation event. (B) The relative significance intensity in different types of misinformation. The relative significance intensity in different types of misinformation showed a unique distribution, so the research needed to consider the differences and common characteristics of the different types of misinformation. CM_neg: number of negative sentiments about communication media and celebrities; CM_non: number of nonnegative sentiments about communication media and celebrities; DOM_neg: number of negative sentiments about disseminators and producers of misinformation; DOM_non: number of nonnegative sentiments about disseminators and producers of misinformation; GA_neg: number of negative sentiments about the official government and public authority representatives; GA_non: number of nonnegative sentiments about the official government and public authority representatives; LG_neg: number of negative sentiments about local government; LG_non: number of nonnegative sentiments about local government; MCB_neg: number of negative sentiments about correction action against misinformation; MCB_non: number of nonnegative sentiments about correction action against misinformation; MD_neg: number of negative sentiments about debunker of misinformation; MD_non: number of nonnegative sentiments about debunker of misinformation; MMO_neg: number of negative sentiments about the main object of misinformation events; MMO_non: number of nonnegative sentiments about the main object of misinformation events; MSB_neg: number of negative sentiments about the spread and manufacturing of misinformation; MSB_non: number of nonnegative sentiments about the spread and manufacturing of misinformation; REP: repost; SP_neg: number of negative sentiments about personal feelings and experience; SP_non: number of nonnegative sentiments about personal feelings and experience; TM_neg: number of negative sentiments about treatment and response measures in misinformation events; TM_non: number of nonnegative sentiments about treatment and response measures in misinformation events.

The overall sample data were regressed at the event level, and the significance of aspect sentiments for each dimension of all events was calculated, as shown in Figure 5. The significance was normalized to obtain the relative significance intensity of sentiments in each dimension. Figure 5 shows that the repost aspect had a significant relationship with public acceptance of misinformation correction. Moreover, a certain degree of regularity in the relative significance intensity of different types of misinformation was revealed. Although there were some differences in the significance of sentiment in each dimension in each event, the results demonstrated that public sentiments in different dimensions were significantly related to whether the public perceived the correction as effective.

We randomly selected two-thirds of the overall sample data for grouped regression based on 4 different types. Table 4 shows the regression results of public acceptance of misinformation correction for various types of misinformation and each corresponding variable. The variables *LIKE*, *LOC*, and *TYPE* were included as control variables but were found to be insignificant across different types. While *FAN* was found to be significant, its coefficient was too small to be considered a reference, and only GEN had a certain impact on public acceptance. Therefore, the influence of gender was always controlled in the grouped regression experiment. In addition, we only considered the causality between public aspect sentiment and public acceptance of correction.

**Table 4:** Regression coefficients for different types of misinformation.

Variable	Type P regression coefficient	Type C regression coefficient	Type G regression coefficient	Type D regression coefficient
MCB_non^a^	1.232^b^	1.168^b^	1.15^b^	1.542^b^
MCB_neg^c^	–0.495^b^	–1.033^b^	–0.816^b^	–0.17^d^
MD_non^e^	1.855^b^	1.183^b^	1.523^b^	1.766^b^
MD_neg^f^	–0.191	–0.652^b^	–0.481	0.428
MMO_non^g^	–0.514^b^	–0.209^b^	–0.798^b^	–0.918^b^
MMO_neg^h^	–1.147^b^	–0.596^b^	–1.746^b^	–0.137
MSB_non^i^	–0.352^b^	–0.24^b^	–0.222^j^	–0.339^j^
MSB_neg^k^	2.011^b^	0.934^b^	1.636^b^	1.606^b^
DOM_non^l^	–0.48^b^	–0.418^b^	–0.477^b^	–0.223
DOM_neg^m^	0.162^b^	0.373^b^	0.103^j^	0.34^b^
CM_non^n^	–0.106^b^	0.255^b^	0.347^b^	0.1
CM_neg^o^	0.723^b^	0.651^b^	0.886^b^	1.693^b^
GA_non^p^	0.574^b^	1.093^b^	0.075	0.872^b^
GA_neg^q^	–0.601^b^	–0.982^b^	–0.915^b^	–0.902^b^
LG_non^r^	–0.042	–0.71^b^	–0.597^b^	0.052
LG_neg^s^	–1.219^b^	–1.46^b^	–1.508^b^	–0.243
TM_non^t^	–0.431^b^	–0.221^b^	–0.5^b^	–0.086
TM_neg^u^	–0.368^b^	–0.384^b^	–0.939^b^	–2.461^b^
SP_non^v^	0.276^b^	1.099^b^	0.347^b^	0.499^b^
SP_neg^w^	–1.064^b^	–0.389^b^	–0.003	–0.316^b^
REP^x^	2.933^b^	2.92^b^	2.729^b^	2.807^b^
GEN^y^	–0.2^b^	–0.017^d^	0.188^b^	0.301^b^
FAN^z^	0^b^	0^b^	0^b^	0^b^
LIKE^a^^a^	0	0^d^	0	0
LOC^a^^b^	–0.001	0.001	0	–0.002
TYPE^a^^c^	–0.003	0	–0.001	0.001
Constant	–0.005	–0.113^b^	–1.052^b^	–1.404^b^

^a^MCB_non: number of nonnegative sentiments about correction action against misinformation.

^b^*P*<.01.

^c^MCB_neg: number of negative sentiments about correction action against misinformation.

^d^*P*<.10.

^e^MD_non: number of nonnegative sentiments about debunker of misinformation.

^f^MD_neg: number of negative sentiments about debunker of misinformation.

^g^MMO_non: number of nonnegative sentiments about the main object of misinformation events.

^h^MMO_neg: number of negative sentiments about the main object of misinformation events.

^i^MSB_non: number of nonnegative sentiments about the spread and manufacturing of misinformation.

^j^*P*<.05.

^k^MSB_neg: number of negative sentiments about the spread and manufacturing of misinformation.

^l^DOM_non: number of nonnegative sentiments about disseminators and producers of misinformation.

^m^DOM_neg: number of negative sentiments about disseminators and producers of misinformation.

^n^CM_non: number of nonnegative sentiments about communication media and celebrities.

^o^CM_neg: number of negative sentiments about communication media and celebrities.

^p^GA_non: number of nonnegative sentiments about the official government and public authority representatives.

^q^GA_neg: number of negative sentiments about the official government and public authority representatives.

^r^LG_non: number of nonnegative sentiments about local government.

^s^LG_neg: number of negative sentiments about local government.

^t^TM_non: number of nonnegative sentiments about treatment and response measures in misinformation events.

^u^TM_neg: number of negative sentiments about treatment and response measures in misinformation events.

^v^SP_non: number of nonnegative sentiments about personal feelings and experience.

^w^SP_neg: number of negative sentiments about personal feelings and experience.

^x^REP: repost.

^y^GEN: gender.

^z^FAN: number of fans.

^aa^LIKE: number of likes.

^ab^LOC: the geographical location of the user’s registration.

^ac^TYPE: user authentication identity type.

All aspects of sentiments in each dimension corresponded to human factors. Therefore, it can be proven that, regardless of the type of misinformation used in this study, public sentiments influenced by human factors affect public acceptance. For SP attributed to internal attribution, it influenced all types. Public acceptance of misinformation correction exhibited a positive correlation with positive public sentiments attributed to personal experience while displaying a negative correlation with negative public sentiments attributed to personal experience. However, its coefficient was not strong in all regression results. On the other hand, for the other dimensions of external attribution, sentiments played a significant role in the regression results. This finding indicated that public sentiments attributed to external attribution had a greater impact on public acceptance than public sentiments attributed to internal attribution regardless of the correction of different types of misinformation.

Regarding correction aspect, public acceptance of misinformation correction was positively correlated with the nonnegative sentiments of *MCB*, negatively correlated with the negative sentiments of *MCB*, and positively correlated with the nonnegative sentiments of *MD*. In addition, the negative sentiments of MD in type C were negatively correlated with public acceptance. For the sentiments of misinformation aspect, the regression coefficients of positive and negative sentiments of the 3 dimensions were integrated. The results supported that public acceptance exhibited a negative correlation with positive sentiments attributed to misinformation while displaying a positive correlation with negative sentiments attributed to misinformation. Regarding the sentiments of communication channels, only in type C and type G public acceptance of misinformation correction was shown to be positively correlated with the nonnegative sentiments of *CM*. However, in all types, public acceptance was positively correlated with the negative sentiments of *CM*. Finally, in the government aspect, public acceptance was only negatively correlated with the negative sentiments of *GA*, positively correlated with the nonnegative sentiments of *GA*, and negatively correlated with aspect sentiments on both of the other dimensions.

### Different Effectiveness of Aspect Sentiments in the Correction of Different Types of Misinformation

To reveal which dimension of sentiments played a greater role in correcting different types of misinformation, it was necessary to compare the regression coefficients of the 4 subsample groups. Therefore, it was necessary to test the difference in coefficients between groups. After pairwise testing among the 4 subsample groups, the results are presented in Table 5. The differences in coefficients between the 2 groups that passed the test are marked with footnotes in the table. Most of these coefficients could be directly compared with the regression coefficients in the grouped regression results.

**Table 5:** Test for differences in coefficients between groups.

Variable	*P* value (type D and type C)	*P* value (type G and type C)	*P* value (type P and type C)	*P* value (type D and type G)	*P* value (type D and type P)	*P* value (type G and type P)
MCB_non^a^	.01^b^	.84	.46	.02^b^	.06^c^	.50
MCB_neg^d^	<.001^e^	.04^b^	<.001^e^	<.001^e^	.008^e^	.006^e^
MD_non^f^	.001^e^	.09^c^	.003^e^	.44	.69	.30
MD_neg^g^	.05^c^	.63	.15	.21	.40	.57
MMO_non^h^	<.001^e^	<.001^e^	<.001^e^	.28	<.001^e^	<.001^e^
MMO_neg^i^	<.001^e^	<.001^e^	<.001^e^	<.001^e^	<.001^e^	<.001^e^
MSB_non^j^	<.001^e^	<.001^e^	.13	.42	<.001^e^	<.001^e^
MSB_neg^k^	<.001^e^	<.001^e^	<.001^e^	.72	<.001^e^	<.001^e^
DOM_non^l^	.13	<.001^e^	.51	.001^e^	.09^c^	<.001^e^
DOM_neg^m^	.81	<.001^e^	<.001^e^	.02^b^	<.001^e^	<.001^e^
CM_non^n^	.15	.22	<.001^e^	.06^c^	.08^c^	<.001^e^
CM_neg^o^	<.001^e^	.001^e^	.12	<.001^e^	<.001^e^	.02^b^
GA_non^p^	.19	<.001^e^	<.001^e^	<.001^e^	.04^b^	<.001^e^
GA_neg^q^	.48	.38	.001^e^	.93	.17	.06^c^
LG_non^r^	<.001^e^	.17	<.001^e^	<.001^e^	.07^c^	<.001^e^
LG_neg^s^	<.001^e^	.81	.28	<.001^e^	<.001^e^	.27
TM_non^t^	.38	<.001^e^	.08^c^	.006^e^	.06^c^	.52
TM_neg^u^	<.001^e^	<.001^e^	.82	<.001^e^	<.001^e^	<.001^e^
SP_non^v^	<.001^e^	<.001^e^	<.001^e^	.18	<.001^e^	<.001^e^
SP_neg^w^	.64	<.001^e^	<.001^e^	.002^e^	<.001^e^	<.001^e^
REP^x^	<.001^e^	<.001^e^	.02^b^	.06^c^	<.001^e^	.009^e^

^a^MCB_non: number of nonnegative sentiments about correction action against misinformation.

^b^*P*<.05.

^c^*P*<.10.

^d^MCB_neg: number of negative sentiments about correction action against misinformation.

^e^*P*<.01.

^f^MD_non: number of nonnegative sentiments about debunker of misinformation.

^g^MD_neg: number of negative sentiments about debunker of misinformation.

^h^MMO_non: number of nonnegative sentiments about the main object of misinformation events.

^i^MMO_neg: number of negative sentiments about the main object of misinformation events.

^j^MSB_non: number of nonnegative sentiments about the spread and manufacturing of misinformation.

^k^MSB_neg: number of negative sentiments about the spread and manufacturing of misinformation.

^l^DOM_non: number of nonnegative sentiments about disseminators and producers of misinformation.

^m^DOM_neg: number of negative sentiments about disseminators and producers of misinformation.

^n^CM_non: number of nonnegative sentiments about communication media and celebrities.

^o^CM_neg: number of negative sentiments about communication media and celebrities.

^p^GA_non: number of nonnegative sentiments about the official government and public authority representatives.

^q^GA_neg: number of negative sentiments about the official government and public authority representatives.

^r^LG_non: number of nonnegative sentiments about local government.

^s^LG_neg: number of negative sentiments about local government.

^t^TM_non: number of nonnegative sentiments about treatment and response measures in misinformation events.

^u^TM_neg: number of negative sentiments about treatment and response measures in misinformation events.

^v^SP_non: number of nonnegative sentiments about personal feelings and experience.

^w^SP_neg: number of negative sentiments about personal feelings and experience.

^x^REP: repost.

Combining the analyses in Tables 4 and 5, we observed that, excluding the effect of repost, in type P, the aspect sentiment with the largest effect was the negative sentiments of *MSB* followed by the nonnegative sentiments of *MD* and the nonnegative sentiments of *MCB*. The negative sentiments of *LG* and the negative sentiments of *MMO* also played a significant role in determining whether the public recognized the correction. This finding suggested that, compared with the correction of other types of misinformation, public sentiments about the spread and manufacturing of misinformation in type P played a more significant role. In type C, the negative sentiments of *LG* were the most influential aspect sentiments, whereas *MCB* and *GA* had large regression coefficients, whether positive or negative. This phenomenon indicated that, in type C, public sentiments toward the correction aspect as well as the government aspect played a more significant role. In type G, the negative sentiments of *MMO* had a more significant effect. Public attention and discussion on the misinformation aspect had a significant impact on the correction of type G. Furthermore, the role of local government in this type of misinformation needed to be considered as public perception of local government also played a significant role. In type D, the most significant impact on misinformation correction was from the negative sentiments of *TM*, with the negative sentiments of *CM* also playing a vital role. Notably, the causality between public acceptance of misinformation correction and negative sentiments of *TM* was apparent. The regression coefficient of negative sentiments of *CM* was also the highest among the 4 types of misinformation. This showed that, for the correction of type D, public discussions about treatment and response measures of the government and communication channels better reflected public acceptance of correction.

### Robustness Test

Finally, to ensure the robustness and reliability of the experimental results and conclusions, the remaining third of the sample data was randomly selected from the entire sample and regrouped according to the 4 different types of misinformation for further testing. The results of the robustness test are shown in Table 6.

The test results showed only minor changes in regression coefficients and significance, which did not alter the overall results and conclusions of the experiment. On the basis of this, it could be concluded that the experimental results of this study were robust and reliable.

**Table 6:** Robustness test results using different data.

Variable	Type P regression coefficient	Type C regression coefficient	Type G regression coefficient	Type D regression coefficient
MCB_non^a^	1.123^b^	1.194^b^	1.153^b^	1.493^b^
MCB_neg^c^	–0.567^b^	–1.02^b^	–0.854^b^	–0.212^d^
MD_non^e^	1.813^b^	1.158^b^	1.615^b^	1.773^b^
MD_neg^f^	–0.307	–0.629^b^	0.056	0.006
MMO_non^g^	–0.51^b^	–0.212^b^	–0.782^b^	–1.024^b^
MMO_neg^h^	–1.148^b^	–0.62^b^	–1.786^b^	–0.065
MSB_non^i^	–0.344^b^	–0.252^b^	–0.375^j^	0.204
MSB_neg^k^	1.941^b^	0.919^b^	1.518^b^	1.574^b^
DOM_non^l^	–0.492^b^	–0.456^b^	–0.483^b^	–0.039
DOM_neg^m^	0.137^b^	0.393^b^	0.1^d^	0.272^b^
CM_non^n^	–0.044	0.231^b^	0.407^b^	0.1
CM_neg^o^	0.705^b^	0.636^b^	0.916^b^	1.638^b^
GA_non^p^	0.556^b^	1.121^b^	0.024	0.653^b^
GA_neg^q^	–0.411^b^	–0.939^b^	–0.826^b^	–1.045^b^
LG_non^r^	–0.096	–0.686^b^	–0.424^b^	0.145
LG_neg^s^	–1.205^b^	–1.687^b^	–1.326^b^	–0.206
TM_non^t^	–0.496^b^	–0.238^b^	–0.515^b^	0.002
TM_neg^u^	–0.398^b^	–0.384^b^	–0.881^b^	–2.421^b^
SP_non^v^	0.266^b^	1.086^b^	0.342^b^	0.471^b^
SP_neg^w^	–1.054^b^	–0.373^b^	0.043	–0.194^d^
REP^x^	2.884^b^	2.906^b^	2.735^b^	2.818^b^
GEN^y^	–0.238^b^	–0.015	0.203^b^	0.325^b^
FAN^z^	0^j^	0^b^	0^b^	0^b^
LIKE^a^^a^	0	0	0	0
LOC^a^^b^	–0.001	0.001	0	–0.003
TYPE^a^^c^	–0.002	0.001	0	0.001
Constant	0.038	–0.12^b^	–1.058^b^	–1.398^b^

^a^MCB_non: number of nonnegative sentiments about correction action against misinformation.

^b^*P*<.01.

^c^MCB_neg: number of negative sentiments about correction action against misinformation.

^d^*P*<.10.

^e^MD_non: number of nonnegative sentiments about debunker of misinformation.

^f^MD_neg: number of negative sentiments about debunker of misinformation.

^g^MMO_non: number of nonnegative sentiments about the main object of misinformation events.

^h^MMO_neg: number of negative sentiments about the main object of misinformation events.

^i^MSB_non: number of nonnegative sentiments about the spread and manufacturing of misinformation.

^j^*P*<.05.

^k^MSB_neg: number of negative sentiments about the spread and manufacturing of misinformation.

^l^DOM_non: number of nonnegative sentiments about disseminators and producers of misinformation.

^m^DOM_neg: number of negative sentiments about disseminators and producers of misinformation.

^n^CM_non: number of nonnegative sentiments about communication media and celebrities.

^o^CM_neg: number of negative sentiments about communication media and celebrities.

^p^GA_non: number of nonnegative sentiments about the official government and public authority representatives.

^q^GA_neg: number of negative sentiments about the official government and public authority representatives.

^r^LG_non: number of nonnegative sentiments about local government.

^s^LG_neg: number of negative sentiments about local government.

^t^TM_non: number of nonnegative sentiments about treatment and response measures in misinformation events.

^u^TM_neg: number of negative sentiments about treatment and response measures in misinformation events.

^v^SP_non: number of nonnegative sentiments about personal feelings and experience.

^w^SP_neg: number of negative sentiments about personal feelings and experience.

^x^REP: repost.

^y^GEN: gender.

^z^FAN: number of fans.

^aa^LIKE: number of likes.

^ab^LOC: the geographical location of the user’s registration.

^ac^TYPE: user authentication identity type.

## Discussion

### Different Types of Misinformation Require Different Correction Communication

The study findings indicated that there were differences in public acceptance of correction among different types of misinformation and the key attributions that affect public acceptance also differed among them. However, regardless of the number of public discussions or the regression results, external attributions were the most significant among the corrections of 4 types of misinformation. The experimental results indicated that personal feelings and experience had an impact but were not the most important. The attributions in the aspects of correction and misinformation most directly influenced public acceptance of correction. While the media and government were the clear targets of most public sentiment responses, public attitudes toward these 2 aspects of attributions were significantly different from those toward other attributions. Compared with existing literature on misinformation correction effectiveness [2], this study provided a new analytical perspective.

In the correction of *prevention and treatment*, the regression results indicated that the public disliked misinformation and expected timely and accurate correction, with the government playing a crucial role. Implementers of correction and clarification of misinformation should be chosen carefully. In the correction of *conspiracy*, the findings illustrated that the public was dissatisfied with both the correction communication method and the implementers. Especially when the government confirmed and corrected misinformation, the public expressed negative sentiments toward local governments more frequently, so the way and timing of government involvement in the correction process needs to be considered prudently. Representatives with greater credibility could play a better role in the correction process [20]. In the correction of *government measures*, public negative sentiments toward misinformation revealed their attention to specific government measures. This showed that, in addition to the negative impact of misinformation, the public also sought to eliminate various inconveniences caused by the pandemic, reflecting their expectations of the government. However, in the correction of *COVID-19 development*, the study found that the public paid more attention to government treatment and response measures. The public considered the impact of treatment measures on themselves or whether the measures met their physical or psychological needs. The spread of this type of misinformation also stemmed from public concern about the uncertainty risk [57].

However, previous studies have emphasized that corrections are different depending on the nature [2,58]. The current approach to correction communication is too simplistic and repetitive, leading to public apathy and backlash [33]. It is necessary to adopt different methods of correcting misinformation to prevent stress reactions or aversion to correction and, ultimately, prevent the public from being exposed to misinformation [34]. Public demand for correction measures varies depending on the differences in misinformation types. The investigation into the specific attributions of public acceptance of misinformation correction is valuable as it uses the aspect sentiment analysis method to not only determine public acceptance but also uncover the underlying attributions affecting it. Ultimately, the fundamental issue is that the public must trust the corrections they receive, which requires flexible and diverse methods of communication and correction channels that the public trusts.

### The Collapse of Media Credibility

The media play a supporting role in public acceptance of misinformation correction, but the effectiveness of the media in this regard is currently questionable. Public distrust of communication channels, particularly the media, was evident across all types of misinformation. Negative sentiments about communication channels were positively correlated with public acceptance of correction, indicating that the media were not perceived as trustworthy by the public. Media failure to gain public trust had resulted in a negative impact on the effectiveness of corrections. Discussions involving authoritative experts and celebrities were the only aspects of communication channels that generated positive sentiments.

In contrast, the public was more likely to trust authoritative experts. Analysis of comments revealed that one of the main reasons for this distrust was the tendency of some media outlets to prioritize speed over verification, leading to the spread of rumors and misinformation. Therefore, the public expressed their distrust of both traditional media and “we-media.” The collapse of media credibility has reduced the persuasive power of correction spread by the media, thereby diminishing the effectiveness of correction. Enhancing the role of communication channels in promoting public acceptance of corrections might require the support of authoritative experts [20]. Therefore, the media need to work toward rebuilding their image in the public impression to regain lost credibility and effectively support the spread and effect of misinformation correction.

### The Crucial Role of the Government

The government plays a crucial role in public acceptance of misinformation correction. Public sentiments toward correction of different types of misinformation also raised significant concerns regarding government involvement. However, experimental findings on public aspect sentiments toward the government demonstrated that only positive sentiments of the *GA* dimension were positively correlated with public acceptance. Both positive and negative sentiments of the *LG* and *TM* dimensions were negatively correlated with public acceptance. This phenomenon suggested that, when assessing public sentiments regarding the government, only the *GA* dimension had a positive guiding effect.

Although the public still maintained trust in the official government image, expectations of the government persisted. Concerning local governments and treatment and response measures, public trust in some local governments was eroded due to the pandemic, aligning with previous research findings [8]. Many treatment and response measures also failed to meet public expectations. Nevertheless, there is no doubt that the government plays a decisive role in both managing the pandemic and mitigating the impact of misinformation on the public [28,59]. Consequently, to enhance communication efficacy and foster public government engagement, trust between the government and the public must be restored. The pivotal role of the government in shaping public acceptance of misinformation correction cannot be overstated as it also exerts a positive influence on other aspects. Nonetheless, based on the results of this study, efforts are needed to solidify the official government image, rebuild trust in local governments, and implement comprehensive response measures to fully leverage the government’s crucial role.

### Limitations and Directions for Future Research

It is important to note that the data used in this study solely originated from user interactions on the Weibo platform. As a result, the analysis of user behaviors and causality can only be applied within the context of Weibo. Therefore, further verification is necessary to generalize the research findings and conclusions to other platforms. Furthermore, the data used in this study specifically pertained to rumors surrounding the COVID-19 pandemic, and they did not encompass rumors unrelated to the pandemic. Consequently, the extension of conclusions and potential variations in other types of misinformation warrant exploration in future research.

Although this study incorporated several control variables and strived to focus on the role of aspect sentiments, it is important to acknowledge that there might be variables associated with personal characteristics that were not accessible within the existing data. This study primarily examined group behavior and might have limitations in accounting for the influence of individual traits. In addition, data on individuals who do not disclose their opinions cannot be obtained, which is a limitation of using social media data for research. As the data were from Chinese social media, more data are needed to verify the expansibility of relevant conclusions to countries with other cultural backgrounds in future research. This study will help identify effective methods for correcting misinformation that are not limited to the pandemic.

### Conclusions

Misinformation correction often falls short in effectively reaching and persuading the public, necessitating a thorough investigation into the reasons influencing public acceptance of such efforts. This study identified crucial sentiment attributions that shape public acceptance of correction regarding different types of misinformation. It highlighted the role of aspect sentiments in analyzing public acceptance of misinformation correction.

The findings revealed that a flexible, issue-specific approach is required for correcting different types of misinformation. The correction strategies also require flexible communication tailored to public sentiment attribution. The decline in media credibility negatively impacted the effectiveness of misinformation correction. Regulating media behavior and rebuilding media image are essential for regaining public trust. Furthermore, the government plays a critical role; in particular, some local governments need to regain the trust of the public, enabling government-led correction communication to be more effective.

This study identified key reasons contributing to the difference in public acceptance of misinformation correction, focusing on public sentiment attributions. It provided an empirical and methodological foundation for addressing misinformation governance to some extent.

## Appendix

Multimedia Appendix 1 Details and types of misinformation-spreading events.

## Abbreviations

ABSA: aspect-based sentiment analysis
BERT: Bidirectional Encoder Representations from Transformers

## Data Availability

The data sets generated during and analyzed during this study are available from the corresponding author on reasonable request.
