# Supplementary material for: Investigation of Public Acceptance of Misinformation Correction in Social Media Based on Sentiment Attributions: Infodemiology Study Using Aspect-Based Sentiment Analysis
Source: J Med Internet Res. 2024 Aug 16;26:e50353. doi: 10.2196/50353 (PMC11364945; doi:10.2196/50353)
Supplement: Multimedia Appendix 1 [file jmir_v26i1e50353_app1.docx]

**Appendix 1.** Details and types of misinformation spreading events

| ID | Type | Details of Misinformation |
| --- | --- | --- |
| NO.1 | Prevention & Treatment | On January 26, 2020, CCTV would broadcast a program in which host Bai Yansong invited Zhong Nanshan to introduce the pandemic. |
| NO.2 | Conspiracy | On January 29, 2020, some netizens disclosed in a video on social media that SF Express couriers opened users’ packages privately by intercepting goods and publicly peddled them in the video. |
| NO.3 | Conspiracy | On January 29, 2020, masks ordered by netizens were seized and confiscated by customs. |
| NO.4 | Government Measures | On January 30, 2020, some netizens questioned the Wuhan Red Cross for selling aid vegetables from Shouguang, Shandong province at low prices through supermarkets. |
| NO.5 | Prevention & Treatment | On January 31, 2020, the reporter learned from the Shanghai Institute of Materia Medica of the Chinese Academy of Sciences that the institute had preliminarily found that Shuanghuanglian oral liquid, a proprietary Chinese medicine, could inhibit the novel coronavirus. |
| NO.6 | Government Measures | On February 4, 2020, it was reported online that the People’s Liberation Army would take over pandemic prevention and control and public security in Wuhan. |
| NO.7 | Prevention & Treatment | On February 4, 2020, Academician Zhong Nanshan received a visit from the boss of a pharmaceutical company in the United States. |
| NO.8 | Prevention & Treatment | On February 5, 2020, academician Li Lanjuan recommended new drugs to prevent COVID-19. |
| NO.9 | COVID-19 development | On February 5, 2020, some netizens said that there was a large outbreak of pandemic in Baibuting community. |
| NO.10 | Conspiracy | On Feb. 7, 2020, a letter claiming to be written by Li Wenliang’s wife went viral. |
| NO.11 | Conspiracy | On February 10, 2020, some netizens posted rumors: After the Jiangsu medical team arrived at Wuhan Airport, supplies were robbed, luggage was lost, and doctors and nurses were transported in local trucks. |
| NO.12 | Conspiracy | On February 10, 2020, online rumors said that the fourth batch of medical workers from Ningbo who rushed to Hubei to help Hubei had not received the medical supplies consigned to Wuhan by plane. |
| NO.13 | Conspiracy | On February 16, 2020, the news spread online that Huang Yanling, a female graduate student at the Wuhan Institute of Viroology, Chinese Academy of Sciences, was the COVID-19 patient zero. |
| NO.14 | Conspiracy | On February 23, 2020, the old news that five foreign athletes were sent to Wuhan Jinyintan Hospital due to imported infectious diseases during the Military Games sparked heated discussion. |
| NO.15 | Prevention & Treatment | On February 25, 2020, Professor Huang Jinhai’s team from the School of Life Sciences of Tianjin University announced that it had successfully developed an oral vaccine for the novel coronavirus. |
| NO.16 | Conspiracy | On March 1, 2020, rumors circulated that Moscow police had violently enforced law, mistreated people in quarantine, and taken away Chinese citizens without reason. |
| NO.17 | Conspiracy | On March 2, 2020, an account of Toutiao published an article that a Ningbo woman donated masks to quickly become a US citizen. |
| NO.18 | Prevention & Treatment | On March 3, 2020, it was announced that Angong Niuhuang Pill can prevent COVID-19. |
| NO.19 | Government Measures | On March 5, 2020, a picture circulated of the “Emergency Notice on the Cancellation of traffic block port in towns and Villages” issued by the Tianmen Headquarters for the Prevention and Control of the novel coronavirus Pneumonia. |
| NO.20 | Government Measures | On March 10, 2020, it was reported online that Beijing residents’ travel was normalized on March 16, public transportation was normalized on March 17, and the city’s first-level response and shift home working mode were ended on March 24, and work resumed. |
| NO.21 | Conspiracy | On March 20, 2020, the writer Fangfang said in her online series of Wuhan diaries that nurse Liang, who helped Wuhan, died. |
| NO.22 | Conspiracy | On June 17, 2020, restaurants in Beijing were closed for 48 hours for nucleic acid testing. |
| NO.23 | COVID-19 development | On June 19, 2020, according to media reports, an elder walked from Bozhou, Anhui Province to Zhejiang province because he did not have a mobile phone and could not show his health code. |
| NO.24 | COVID-19 development | On June 22, 2020, it was revealed that the cargo on a SF Airlines flight to Changsha was positive for COVID-19. |
| NO.25 | COVID-19 development | On June 24, 2020, Beijing 301 Hospital was currently infected, and more than 100 closed communities were added to Haidian. |
| NO.26 | COVID-19 development | On December 8, 2020, Chengdu University of Technology was closed due to the local pandemic in Chengdu. |
| NO.27 | Conspiracy | On March 20, 2022, a chat record of “black Express” fabricating nucleic acid in order to make money spread among group chats. |
| NO.28 | Government Measures | On April 8, 2022, there were rumors that Shanghai was about to begin militarized governance and that armed police forces would take over the city’s communities. |
| NO.29 | Conspiracy | On April 10, 2022, a video circulating online showed quarantined personnel sleeping in cardboard boxes in Baoshan square cabin, Shanghai. |
| NO.30 | Conspiracy | On April 10, 2022, a netizen claimed that life detectors have been used at all expressway exits in Shanghai to prevent people from escaping from the city from being hidden in trucks. |
| NO.31 | Conspiracy | On April 14, 2022, Qian Wenxiong, the director of a Shanghai center, committed suicide and then his wife did the same. |
